# Physicochemical and Sensory Characteristics of Sausages Made with Grasshopper (*Sphenarium purpurascens*) Flour

**DOI:** 10.3390/foods11050704

**Published:** 2022-02-27

**Authors:** Salvador O. Cruz-López, Yenizey M. Álvarez-Cisneros, Julieta Domínguez-Soberanes, Héctor B. Escalona-Buendía, Claudia N. Sánchez

**Affiliations:** 1 Departamento de Biotecnología, Universidad Autónoma Metropolitana, Iztapalapa, Av. Ferrocarril de San Rafael Atlixco 186, Col. Leyes de Reforma 1a. Sección, Alcaldía Iztapalapa, Ciudad de México 09310, Mexico; springrar@gmail.com (S.O.C.-L.); hbeb@xanum.uam.mx (H.B.E.-B.); 2 Escuela de Dirección de Negocios Alimentarios, Universidad Panamericana, Josemaría Escrivá de Balaguer 101, Aguascalientes 20296, Mexico; jdominguez@up.edu.mx; 3 Facultad de Ingeniería, Universidad Panamericana, Josemaría Escrivá de Balaguer 101, Aguascalientes 20296, Mexico; cnsanchez@up.edu.mx

**Keywords:** sausages, edible insects, grasshopper, protein, starch chewy, non-meat ingredients

## Abstract

Insects are currently of interest due to their high nutritional value, in particular for the high concentration of quality protein. Moreover, it can also be used as an extender or binder in meat products. The objective was to evaluate grasshopper flour (GF) as a partial or total replacement for potato starch to increase the protein content of sausages and achieve good acceptability by consumers. GF has 48% moisture, 6.7% fat and 45% total protein. Sausages were analyzed by NIR and formulations with GF in all concentrations (10, 7, 5 and 3%) combined with starch (3, 5 and 7%) increased protein content. Results obtained for the sausages formulations with grasshoppers showed an increase in hardness, springiness, gumminess and chewiness through a Texture-Profile-Analysis. Moreover, a* and b* are similar to the control, but L* decreased. The check-all-that-apply test showed the attributes highlighted for sausages with GF possessed herbal flavor, brown color, and granular texture. The liking-product-landscape map showed that the incorporation of 7 and 10% of GF had an overall liking of 3.2 and 3.3, respectively, considered as “do not like much”. GF can be used as a binder in meat products up to 10% substitution. However, it is important to improve the overall liking of the sausage.

## 1. Introduction

The FAO has suggested that the only way to end hunger is with the incorporation of insects into the diet. There are two suggested ways for their consumption: eating them as a sole dish or as an ingredient incorporated in a food product. Insects are an essential source of high-quality proteins and are considered similar to those present in meat and fish, but at a lower price [1]. Insects, as a group, represent the most significant biomass of the planet. Their total weight is more in quantity than the sum of the weight of all animals in total. In addition, in every ecosystem, they constitute an important animal protein. Their nutritive value converts them into a complex food, and their body mass is composed in a range of 20–70% of proteins, depending on their state of maturity and their polyunsaturated fat content; therefore, it is possible to compare them with the nutritional value of chicken, beef or pork [2].

There has been an increased tendency to study the functional properties of the molecules present in edible insects. One of the uses of this type of molecule is additives in the food industry as food preservatives, antioxidants, protein isolates, and extenders or meat binders [1,2,3]. They can be incorporated into the human diet due to their high nutritional value. In addition, their production can trigger job opportunities in the rural communities where they are produced naturally [4], which contributes to the sustainability of the planet [3]. Moreover, their use can provide food security around the world [5].

Over 3000 ethnic groups in African, Asian, and Latin American countries eat insects as part of their regular diet [1,6]. Insects represent approximately 60% of global biodiversity; however, it is thought that only 20% has been described [7]. The most consumed insects around the world are beetles (Coleoptera) and caterpillars (Lepidoptera), which represent approximately 50% of the total. In contrast, the rest are represented by crickets, locusts, grasshoppers (Orthoptera), ants, bees, wasps (Hymenoptera), termites (Isoptera), and flies (Diptera) [3,7]. From the Orthoptera order, the most common reared insects are crickets, such as *Acheta domesticus* (house cricket), *Gryllodus sigillatus* (banded cricket), *Gryllus assimilis*, *Gryllus testaceus*, *Gryllus bimaculatus* (field crickets), grasshoppers *Locusta migratora* (migratory locust) and *Sphenarium purpurascens* [5,7]. Mexico has vast biodiversity of insects with a total of 300 to 550 species, all of which are considered to have high nutritional value, which is incorporated empirically from the gastronomic point of view to different traditional dishes [6,7]. Within Mexico’s varied and complex cuisine, the use of edible insects dates back to Mexico’s indigenous origins. The most popular edible Mexican insect is *Sphenarium purpurascens* (SP) known as “grasshopper” or “red grasshopper”, commonly called “chapulin”, it belongs to the order of Orthoptera, Family Pyrgomorphidae—the term itself is specific to Mexico and is derived from the Nahuatl word, *chapolin* [6,7,8]. 

Mexican cuisine is considered an intangible heritage of humanity, rich in history, traditions, and ingredients. Not surprisingly, Mexico developed a dining concept that was presented in London in 2015 by kitchen theory, where they incorporated insect matters (mainly powdered but also the whole product) into different dishes designing a multi-sensory experience to introduce insects creatively in order to indulge the foodies into the cultural history of the use of insects [9]. 

There are few reports on the functional properties of *Sphenarium purpurascens* (SP) as food ingredients [10]; almost all of them have focused on the nutritional assessment of the insect. Some authors indicated that 100 g of SP has a total content of macronutrients of 52.74 to 75.87 g protein (51.85 mg essential amino acids, 52.14 mg non-essential amino acids), 6.02–11.0 g lipids, and 15.59–33.17 g carbohydrates. Moreover, grasshoppers can be viewed as a very good source of phenylalanine (22 to 117 mg/g protein), amino acid that plays a vital role in several biochemical processes, including the synthesis of neurotransmitters, thyroxine and melanin [11]. In addition, SP have the following micronutrients: 34.61 mg sodium, 1028.80 mg potassium, 200.95 mg calcium, 17.84 mg zinc, 13.33 mg iron, 123.93 mg magnesium and vitamins B1-3 (0.27 mg thiamin, 0.59 mg riboflavin and 1.56 mg niacin). Having in its content: 2.5–6.28 g ashes, 3.89–11.04 g crude fiber, with a total energetic content of 391.7 kcal [1,7,10,11,12,13]. 

Grasshoppers are considered a high-value ingredient [7,10,11,12,13], because they stand out due to their nutritional properties when considering their high content of protein when compared to other sources of animal origin, complying with the FAO requirements. On the other hand, insects could be used as a texturizing agent in food products due to their techno-functional properties such as water absorption capacity, oil absorption capacity, emulsion stability and gelification capacity [14,15]. They are making it necessary to study other uses, not limited to the most common usage, a ready-to-eat fried version of grasshoppers (chapulin).

Nowadays, binders are used to better technological properties in charcuterie products, preventing water loss because they increase the emulsification and gelification of fat, not contributing to the product’s nutritional value [16]. The most used binders in the meat industry are flour and starch. Therefore, the use of grasshoppers incorporated into flour for further use in meat products as a binder is not out of the question, with an increase in the quality of the consumer’s diet. Taking this into account, the objective of this research was to evaluate the use of grasshopper (*Sphenarium purpurascens*) flour as a partial or total replacement for potato starch (meat binder) in sausages, to increase protein content without reducing the physicochemical properties of the product, and achieving good acceptability by consumers.

## 2. Materials and Methods

### 2.1. Experimental Design

In order to evaluate the effect of grasshoppers (*Sphenarium purpurascens*) flour (GF) incorporated in sausages as a substitute for potato starch (PS), the following design was carried out. For the characterization of grasshopper flour, two batches were obtained and the response variables analyzed were moisture, fat, protein, and CIELab* parameters. Each one of them was evaluated by quadruplicate (*n* = 8). Once the flour was characterized, five formulations with two replicates were made of each batch of GF (*n* = 20). Different mixtures of GF and PS were accomplished in the formulation maintaining a 10% ratio between them (Table 1). Each formulation was analyzed by near-infrared analysis, texture-profile analysis, color, and total protein in triplicate (*n* = 12). All of the results were reported as the mean with its standard deviation. Finally, the sensory evaluation was applied to the formulation that presented the best texture properties.

### 2.2. Preparation of Sphenarium purpurascens’s Flour

*Sphenarium purpurascens* (SP) seasoned with salt and lemon, in a ready-to-eat presentation, were purchased from an exotic meat market in Mexico City named “San Juan” and were refrigerated at 4 °C until used. The grasshoppers were cleaned of foreign matter. Later, they were dried in an oven (Felisa model FE-292AD, Jalisco, Mexico) at 70 °C for 8 h. They were subjected to a size reduction to obtain a powder, which was sieved in a No. 40 mesh. Two batches were obtained and labeled as grasshopper flour (GF) stored for later characterization. The moisture (method 950.46B), fat content (method 960.69) and protein content (method 981.10) were determined according to the Association of Official Agricultural Chemists (AOAC) guidelines [17]. The color was determined using a previously calibrated colorimeter (ColorFlex EZ 45/0 (HunterLab, Reston, VA, USA, EE. UU.), with a 19.1 mm aperture, Illuminate D65 and 10° standard observer. The determinations were carried out in quadruplicate. The parameters measured were CIELab* [18]. 

### 2.3. Sausage Preparation

Five different sausage formulations were prepared with GF and control according to Table 1. Lean pork was purchased from a local market, removing visible fat and connective tissue. The meat was ground in a Moulinex DPA2 Food Processor (Moulinex, Ecully, France) and mixed with sodium chloride, sodium nitrate (curing salt) and Hamine^®^ commercial phosphate mixture (McCormick-Pesa, México City, Mexico), incorporating half of the total ice in one min. Frozen lard (pork back fat) was added and emulsified for one more minute. To compensate for fat and sodium reduction in the formulation, ice was used to adjust to 100% of added water. The rest of the ice was added and emulsified during 2–3 min until there was total ingredient incorporation, taking care to maintain the meat batter temperature (12 ± 2 °C). The GF and PS were added with the different salts. Meat batters were stuffed into a 20-mm cellulose casing and cooked into a water bath until reaching an internal temperature of 70 ± 2 °C for 30 min, cooled in an ice bath, and vacuum packaged employing a vacuum machine (EVD4 Torrey, México City, Mexico) in oxygen-impermeable bags. 

### 2.4. Analysis of Texture and Color

Texture profile analysis (TPA) was evaluated through a texture analyzer (Brookfield model CT3, AMETEK, Berwyn, PA, USA) equipped with a cylindrical probe. Sausage samples were cut into 20-mm length cylinders and axially compressed to half of their original height in two consecutive cycles, with a constant crosshead speed of 2 mm/s and a waiting period of 5 s between them. Texture profile parameters were calculated from the force-time deformation curves. Several parameters were calculated. Hardness is defined as the maximum peak force during the first compression. Cohesiveness refers to the extent that the sample can be deformed before rupture (A2/A1), where A2 is the positive force area during the second compression, and A1 refers to the first one. Gumminess, described as the force required to break up the sample to make it ready for swallowing, is obtained by multiplying hardness times cohesiveness. Chewiness refers to the work required to chew a solid sample until a state of swallowing, and it is obtained by multiplying hardness times cohesiveness times springiness. Finally, springiness is defined as the ability of the sample to recover its original form after the deformation force has been removed, and it is calculated by the height between the end of the first compression and the beginning of the second one [19]. 

Sausage samples were cut transversely with 25 mm thickness, and color was determined utilizing a colorimeter (ColorFlex EZ spectrophotometer 45/0; HunterLab, Reston, VA, USA) previously calibrated. The CIELAB color coordinates L* (lightness), a* (redness), and b* (yellowness) were set at a 10° angle observer and D65 light source. The average color per sample was determined from three readings by rotating the sample 90 ° [18]. 

### 2.5. Near-Infrared Analysis

Samples bags of 20 g were introduced in a petri dish (NIR11055058), with the size of 82 mm diameter and 25 mm high, the material of it is made of silicon rubber and high resistance glass, which is unique for the near-infrared equipment. FT-NIR Buchi NIRMaster, Flawil, Switzerland (NIR) is a spectrophotometer with a wavelength ranging 800–2500 nm; the obtained spectra was integrated and analyzed through a library (NIRN555-502, 1000242776) that is used specifically for sausage products. The outcome of the use of the library gives us the parameters, which are: moisture, fat, protein, ashes, salt, water activity (aw), bioavailable protein (BEFFE) that is obtained by subtracting protein to protein of connective tissue, monounsaturated fatty acids (MUFA), polyunsaturated fatty acids (PUFA), non-protein nitrogen (NPN) and saturated fatty acids (SFA). Four sausages of the same formulation were analyzed by triplicate, obtaining 12 results.

### 2.6. Sensory Evaluation

The sensory evaluation was applied to the formulation with the best texture properties with the grasshopper: the formulation that only used grasshopper (FI) and the mixture of grasshopper-starch formulation (F2). The latter was compared with the control formulation (starch) and commercial sausage. The sensory analysis was done by consumers (*n* = 100) between 19 and 40 years old. The session was organized in two steps. In the first one, participants were given four portions simultaneously of different formulations of sausages cut in 20-mm length. Consumers were instructed to cleanse their palates between samples using crackers and water. The sausages were evaluated according to general liking [20,21] using a seven-point hedonic horizontal scale, from “Dislike a lot” (1) to “Like a lot” (7). During the second step, a check-all-that-apply (CATA) test was applied, in which they chose from a list of 37 attributes related to taste, smell, texture, and appearance of the descriptors that apply to the sample [22,23].

### 2.7. Statistical Analyses

Statistical analysis was performed using XLSTAT software Version 2014.5.03 (Addinsoft, Paris, France) with statistical significance determined using an alpha value of 0.05. The results were analyzed using a one-way analysis of variance (ANOVA), and Tukey means comparison tests between the treatments for each of the methodologies used. 

For the sensory tests, the frequency of each sensory attribute was determined by counting the number of consumers that used that term to describe each sample, and factorial correspondence analysis (FCA) was used to get a bidimensional representation of the samples and the relationship between samples and terms from the CATA data. Friedman’s non-parametric test and frequency distribution tests for the degree of liking were performed. In addition, Liking Product Landscape (LPL) was used to map hedonic evaluations of consumers [21].

## 3. Results

### 3.1. Characterization of the Flour of Grasshopper

Fine flour was obtained from edible grasshoppers that have 48 ± 0.35% moisture, 6.7 ± 0.5% fat, and 45 ± 1.2% total protein. Color tends to red (a* = 13.93 ± 0.25) and yellow (b* = 21.57 ± 0.35) with an intermediate luminosity (L* = 44.59 ± 0.45). The color values obtained in the CIE a*parameters may be due to the roasting of *S. purpurascens* for consumption, which may promote Maillard reactions due to the presence of amino acids, sugars and proteins causing darkening of the grasshoppers [24].

### 3.2. Texture and Color Analysis

Texture and color analysis are presented in Table 2. The formulation with higher hardness is F2, not finding significant differences within the samples F1 and F4. However, all the formulations except F1 are significantly different from the control, being the least hard. We can observe a significant difference from the control regarding springiness, gumminess, and chewiness. Regarding springiness, there is no significant difference among formulations. For cohesiveness, all formulations are not significantly different between them. Thus, incorporating different concentrations of GF does not affect the properties of springiness and cohesiveness. All the formulations have a greater gumminess and chewiness when they are compared to the control one, being F2 and F4 the ones that present the higher values.

The lightness (L*) of sausages decreases when the insect flour is added, but no significant difference among formulations is appreciated, even though GF concentration decreases. However, all the formulations have significant differences (*p* > 0.05) when compared with the control, and the samples are darker. The parameters of color CIE b* of the different formulations do not have significant differences (*p* < 0.05) between the formulations and the control. However, a* F1 and F2 formulations are not significantly different compared to the control. 

### 3.3. Near-Infrared Analysis of Sausages

There has been an increase in more accurate and faster analysis methods in foods. Therefore, the use of Near-Infrared Analysis (NIR) has been used as an additional tool for proximate measures with good results. On the one hand, it does not employ reagents and it can determine multiple parameters at the same time. A revision has been developed for this technique to predict the chemical composition, quality, and sensory attributes of meat products, showing that NIR is an excellent tool to precisely estimate the chemical composition in seconds [25,26]. We can observe in Table 3 the results obtained by NIR for the different formulations analyzed. In general, we can visualize that GF flour has an influence on the parameters evaluated; all formulations with GF (F1–F4) have significant differences (*p* < 0.05) when compared to the control, except for the fat, aw and NPN parameters. Regarding fat, there is no significant difference between the control, F1 and F3. For aw does not present significant differences (*p* > 0.05) among all formulations and control. With respect to NPN, there is no significant difference between the control and F4. All formulations have a higher percentage of protein compared to the control. Thus, the protein concentration and BEEFE increase with an increment in GF percentage in all formulations. The formulation F1 presented the higher values of fat, protein, ashes, salt, BEEFE and NPN, which indicates the relationship of the incorporation of GF as a substitute for starch.

However, the results obtained of PUFA and MUFA are not higher than the control, and they decreased when grasshopper flour were added to formulation; so, the incorporation of the grasshopper (chapulin) does not affect the value of unsaturated fatty acids in the final product, although they can be present in low concentrations. On the one hand, SFA is associated with inflammatory markers and adipokines [27] if intake is higher than the established recommendation, which could lead to cardiovascular diseases. Our results show that when grasshoppers are included in the formulations, the percentage of SFA decreases. Sodium plays an important role in the regulation of blood pressure, water transport within the cells, and transmission of nerve impulses. Still, excessive intake is linked to hypertension and other cardiovascular diseases. The U.S. dietary guidelines establish that the recommended daily sodium intake should be 23,000 mg as a maximum limit and approximately 21% of the total corresponds to meat and meat products intake. We can observe in Table 2 that the values of salt increase when including grasshoppers in the formulation; this could be explained because when acquired in the market, they were sold with salt [28].

### 3.4. Sensory Analysis

**Figure 1:**
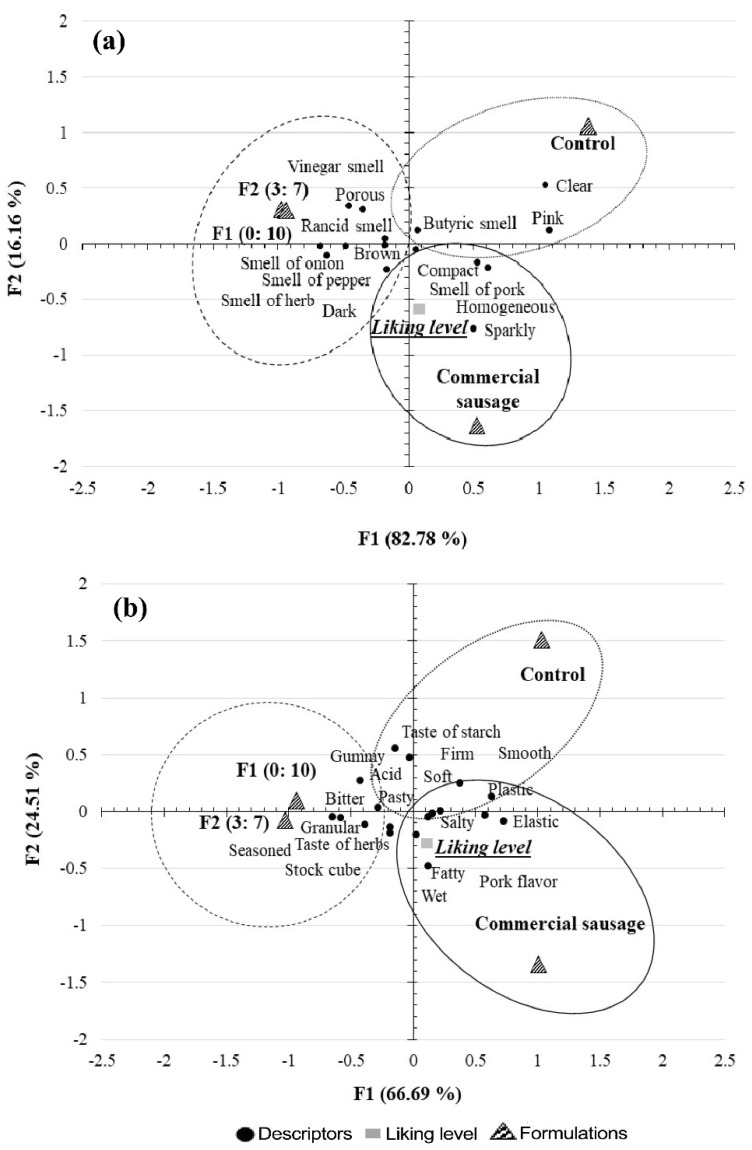
Correspondence factorial analysis of the sausage descriptors. (**a**) smell-appearance, F1 and F2 axes explain 98.94% of the data; (**b**) taste-texture. The F1 and F2 axes explain 91.2% of all the data. Control: sausages formulated with 10% starch without GF; F1 sausages formulated with 10% GF without starch; F2 sausages formulated with 3% starch and 7% GF.

All the formulations with *Sphenarium purpurascens* had better textural characteristics than the control, which means that GF could be a substitute for starch in the manufacturing of sausages. However, the sensory analysis was realized with the formulation of 7 and 10% of grasshopper flour; it is important to know the acceptability of the consumers to a novel product made with a high concentration of a traditional insect from Mexico and as a value-added product because of the nutritive properties of the grasshopper. Sensory analysis techniques are essential to establish the quality of products and to understand consumer preferences [21]. The correspondence factorial analysis (FCA) of sensory descriptive data obtained by CATA explained a 98.94% and 91.20% relationship between the samples and the sensory descriptors of smell-appearance (Figure 1a) and taste-texture (Figure 1b), respectively. Figure 1 shows that the descriptors for the formulations F2 and F1 are in the same cluster for both FCA and the attributes that were pointed out are: vinegar smell, the smell of onion, smell of herb, rancid smell, smell of pepper, porous, brown, dark, acid, pasty, gumminess, taste of herbs, seasoned, stock cube, bitter and granular. It’s important to remember that the formulation does not have spices, therefore the descriptors identified are associated with the GF. On the other hand, the descriptors for the control formulation are clear, pink, butyric smell, firm, soft, smooth and plastic; and for the commercial sausages are bright, compact, with a smell and taste of pork, homogeneous, salty, grease and elastic.

**Figure 2:**
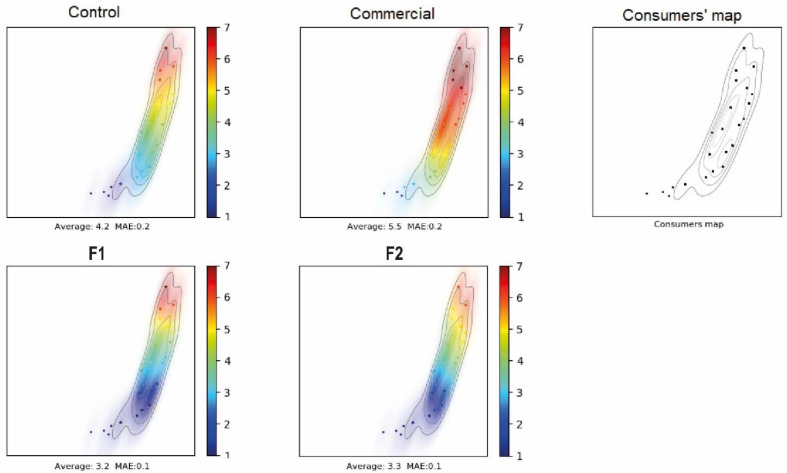
Liking Product Landscape. Consumers’ map created with multidimensional scaling (MDS) fed with overall liking and product acceptance maps created with support vector machines (SVM). Each map presents the average of liking in addition to the mean absolute error (MAE) of the product acceptance maps on a scale of 1 to 7. Control sausages formulated with 10% starch without GF; F1 sausages formulated with 10% GF without starch; F2 sausages formulated with 3% starch and 7% GF.

The hedonic test LPL was used for making a graphical mapping to compare products based on consumer evaluations. LPL can be used to identify consumer preferences. As observed in Figure 2; commercial, control, F1 and F2 were compared. Formulations F1 and F2, which incorporated grasshoppers and that had an overall liking of 3.3 (“do not like much”), are statistically different with respect to the control that had a level of overall liking of 4.2 (“Do not like or dislike”). However, the commercial product had an overall acceptance of 5.5. It can be observed in Figure 2 that the redder the figure, the higher the overall liking. We can observe that the commercial samples were liked the most, followed by the control, and lastly, we can find F1 and F2 formulations. From maps, it can be observed that consumers that do not like “too much” the commercial product are the same as those who do not like the proposal formulations. It could be explained because they do not feel comfortable with the introduction of grasshoppers into their food. Additionally, data density shows that there are no evident clusters of consumers with different global behavior on the samples’ acceptance.

## 4. Discussion

*Sphenarium purpurascens* is a grasshopper widely consumed in southern Mexico in a roasted and seasoned form, and it is customary to eat the entire body. Despite this, many consumers have neophobia due to its appearance despite its high nutritional value. Therefore, incorporating this insect into food in the form of flour can generate products with a high added value due to their high protein content. For all the above, it is important to characterize the flour obtained from SF, since there is very little information reported about the use of *S. purpurascens* in the food industry. In addition, no information has been found in the bibliography on SF for ready for consumption products. Also, cooking methods, like roasting, can also influence its properties. The moisture values are below the ones reported for other insects such as larvae *Tenebrio molitor* 63.18%, *Apomecyna parumpunctata* 59.4%, *Imbrasia epimethea* 79.8%, *Pseudantheraea discrepans* 72.2% and *Imbrasia oscura* 83.0% [29,30,31] but they are similar to another grasshopper, *Sphenarium histrio* (37.04%), *Melanoplus femrrubrum* (39.82 %), *Sphenarium purpurascens* (41.44 %) and *Schistocerca* spp. (43.19%) [11]. According to Melo-Ruiz et al. [11], the difference between the values could be explained by the season in which they were collected (that could be attributed to rainy or dry weather) and the storage conditions of grasshoppers (because they could absorb water from the environment). Although SF is consumed roasted, traders keep it in refrigerated and/or frozen conditions, which can allow the absorption of moisture from the environment and thus can explain why its values are similar to other unprocessed grasshoppers. 

Insects are considered food products high in protein because they contain large amounts of essential amino acids. SF has a higher content of isoleucine, leucine, lysine, methionine, cysteine, phenylalanine, tyrosine, threonine, valine and histidine than that of beef, pork, lamb, chicken, turkey or fish [7], which mean that its protein is highly bioavailable [13]. The protein content and fat of *Sphenarium purpurascens* is within the reported range by other authors that established 52.74–75.87 g protein and 6.02–11.0 g lipids/100 g dry matter for grasshoppers [7,10,11,12,13]. In general, the order of Lepidoptera (caterpillars) and Orthoptera (grasshoppers, locusts, and crickets) are the ones that exhibit a major content of protein [30]. The difference in the parameters of moisture, fat, and protein between the same species of insect, could be attributed to factors such as the period of growth, diet, climate and place of collection of the insects [13,32]. Not all insects are safe to eat, some insects are not edible or cause allergic reactions [14]. It is important to keep in mind that the consumption of insects can cause health risks due to agricultural practices such as residual pesticides and heavy metals, parasitic association and allergic response of sensitive individuals that cannot be ignored [12]. However, one solution to this problem could be the production of these grasshoppers in controlled conditions (farm rearing) where feed could be monitored in order to ensure food safety and the manual harvesting of insects. In southern Mexico, SF is considered an edible insect that is harvested manually in corn and alfalfa fields and this practice is the source of income in many rural areas. 

Insect flour can be used as a meat additive since its techno-functional properties, such as emulsifying stability, allow a high water and oil retention capacity [12,14]. Sausages were obtained using GF as a substitute for PS and their parameters of texture and color were determined. Hardness, springiness and cohesiveness are primary mechanical parameters that can be widely used to characterize the texture properties, sensory attributes and rheological properties of various foods [19,33]. With respect to hardness, chewiness and gumminess, similar results are reported in meat batter (sausages) when using house cricket *Acheta domesticus* flour at 5% and 10% levels [34]. In addition, when using silkworm pupae (*Bombyx mori*) at levels of 5, 10 and 15% [35] and untreated, defatted and acid-hydrolyzed mealworm larvae (*Tenebrio molitor*) and silkworm pupae (*Bombyx mori*) flours at 10% level [36]. On the other hand, the hardness is directly proportional to chewiness and gumminess. Chewiness is the work needed to compress a solid sample to a steady state of swallowing [19]. The results, which means that the GF alone or in combination with the PS presents a good techno-functional property because in all the formulations the hardness did not decrease compared to the control. Lastly, formulations F2 (7 GF: 3 PS) and F4 (3 GF: 7 PS) present the highest values of texture properties; both formulations have de same proportion entre GF and PS with which it can be inferred that both ingredients have a synergistic effect, since they increase their textural properties in combination. Perhaps the protein, which contains the GF, have water retention capacity similar to the meat proteins and binders, improving the cohesion of the particles of different ingredients into the sausages, due that the hydrophobic amino acid towards the fat globule and the hydrophilic amino acids into the aqueous phase [16]. 

The color of food products is one of the principal parameters of which consumers choose the product. Thus, it is important that the additives used do not cause significant color changes in relation to the conventional product [37]. GF tends towards red (a*) and yellow (b*) in darker tones (L*). These results show that the grasshopper flour contributes to color; these findings could consider this ingredient as a pigment. Insect flour can modify color parameters by obtaining darker sausages, usually the edible insect protein is brown or dark in color, which can be attributed to melanin pigments [38]. The results are in accordance with other authors who incorporated grasshopper *Sphenarium purpurascens* [10], cricket *Acheta domesticus* [18,37], yellow worm *Tenebrio molitor L*. [36,38,39] and silkworms (*Bombyx mori*) [35,36] to food products as snacks, paté or meat batter, all the case obtained products darker than the control.

Due to the nutritional and economic properties that SF represents, it is important to know the behavior of this flour incorporated in processed foods. In the particular case of sausages, these are a product that is not considered healthy, but it is an easily accessible food for consumers. The food industry uses different types of binders or extenders to reduce the final costs of the product, regardless of the reduction in the nutritional value of the finished product. The incorporation of GF is reflected in an increase in protein content. Other authors that incorporated *Acheta domesticus* cricket powder to meat emulsion [34] and pork pâté [37], *Bombyx mori* [35] and *Tenebrio molitor* and pupae of silkworms in sausages [39]. The increasing of protein in sausages with GF allows us to offer consumers a product with added value, because the sausage could contain not only enough protein and amino acids but also sufficient non-protein energy to permit the optimal use of dietary protein [11]. On the other hand, it is reported that the protein of SP contains high levels of phenylalanine, glycine, tyrosine, leucine and isoleucine, which together with the rest of the amino acids present, cover the daily requirements of human adults and preschool-aged children [7]. 

NIR can determine the fatty acid content which has a direct relation with human health. Grasshoppers have a desirable fat composition, due to the high level of polyunsaturated fatty acids (PUFAs), SF have reported a higher concentration of PUFA (69.3%) such as linoleic (18:2n-6) and α-linolenic (18:3n-3), compared to 30.6% saturated fatty acids (SFA), that makes it a possible source of high-quality oil [13,40] due to these fatty acids that act as precursors for the synthesis of long chains of polyunsaturated fatty acids such as arachidonic acid, eicosapentaenoic acid (EPA) and docosahexaenoic acid (DHA) [3]. It has been reported that the intake of MUFA and PUFA reduces cholesterol levels, coronary heart disease, inflammatory and immune disorders; therefore, their incorporation in diet can be used as a prevention of diseases [41].

Finally, it is important to know the liking of the consumers regarding a widely consumed product added with a grasshopper flour. There are no descriptors reported by *Sphenarium purpurascens,* but the descriptors of the cricket *Acheta domesticus* incorporated to foods are dark, brown color, hard, gritty, grainy, nutty, pet food or woody flavors, the smell of seeds and grease [42,43,44]. The descriptors: granular, brown, smell of herb and dark are similar between both insects, maybe because they belong to the order Orthoptera, both have chitin within their structure which could give the sensation of grainy, and they have similar feed which is related to the taste and smell to herb. The results obtained are in accordance with other authors who observed that the incorporation of insects into food product decreases the liking level of consumers [4,37]. 

The consumer acceptability of the sausages with GF was lower; this may be due to neophobia, because at the beginning of the test, some consumers tasted the products with a certain rejection of the grasshopper. However, other consumers who have already experienced the flavors and textures of grasshopper made very good evaluations of the product. On the other hand, some authors said that people are usually neophobic towards insects for fear of the unknown, so improving the acceptability of the consumers for this type of product is important. Some strategies are informing consumers about the nutritional properties of insects and improving the image of foods processed with edible insects to establish and increase consumer acceptance [12,14].

## 5. Conclusions

Grasshopper (*Sphenarium purpurascens*) flour could be incorporated in a cooked meat product as a meat binder. Considering the texture parameters, we can consider it better than the control with starch. In addition, grasshopper flour also improves the added value of sausages from the nutritional point of view by the quantity and quality of protein that grasshoppers can contribute to the food. On the other hand, the grasshopper flour provides color to the sausage without the incorporation of synthetic dyes, so it can also have the function of pigment. However, despite all the advantages that the use of grasshopper flour (GF) as a binder could have, the sausages did not have good acceptability, so it can be proposed for a group of consumers who are familiar with the texture, smell and taste of the grasshopper (chapulines) or for consumers willing to try new products. Finally, this kind of product could be categorized as a gourmet product with a high added value.

## Figures and Tables

**Table 1:** Sausage formulations with grasshopper powder.

Ingredients (%)	Formulations ^1^				
	Control	F1	F2	F3	F4
Pork meat	50	50	50	50	50
Frozen lard	15	15	15	15	15
Sodium chloride	2	2	2	2	2
Sodium nitrate	0.3	0.3	0.3	0.3	0.3
Phosphate mixture Hamine^®^	0.5	0.5	0.5	0.5	0.5
Potato starch (PS)	10	0	3	5	7
Grasshopper flour (GF)	0	10	7	5	3

^1^ Ice (frozen water) was employed to complete 100%.

**Table 2:** Textural attributes and color parameters of sausages formulated with grasshopper flour.

Parameters	Formulations ^1^				
	Control	F1	F2	F3	F4
Hardness (N)	14.44 ± 0.97 ^A^	17.71 ± 1.29 ^A,B,C^	21.95 ± 1.1 ^C^	17.19 ± 2.74 ^B^	20.54 ± 1.07 ^B,C^
Springiness (mm)	2.19± 0.13 ^A^	3.22 ± 0.35 ^B^	3.35 ± 0.04 ^B^	3.32 ± 0.11 ^B^	3.41 ± 0.05 ^B^
Cohesiveness	0.83 ± 0.01 ^A^	0.74 ± 0.09 ^A^	0.73 ± 0.02 ^A^	0.82 ± 0.12 ^A^	0.72 ± 0.01 ^A^
Gumminess (N)	8.39 ± 0.71 ^A^	13.23 ± 0.88 ^B^	15.59 ± 0.39 ^C^	14.37 ± 0.64 ^BC^	15.06± 0.29 ^C^
Chewiness (mJ)	18.38 ± 0.42 ^A^	43.16 ± 0.87 ^B^	52.22 ± 0.65 ^D^	45.64 ± 0.39 ^C^	51.43 ± 0.45 ^D^
L*	37.45 ± 0.83 ^C^	26.33 ± 0.81 ^A^	28.01 ± 3.03 ^A,B^	30.48 ± 0.82 ^A,B^	33.46 ± 1.74 ^B,C^
a*	11.02 ± 0.13 ^B^	10.35 ± 0.64 ^B^	10.60 ± 0.29 ^B^	8.21 ± 0.20 ^A^	8.84 ± 0.27 ^A^
b*	11.08 ± 0.12 ^A^	12.17 ± 0.92 ^A^	12.12 ± 0.38 ^A^	12.37 ± 0.24 ^A^	11.45 ± 0.46 ^A^

^1^ All values are mean ± standard deviation of three replicates (*n* = 12). Different letters in the same row means significant differences between formulations at *p* < 0.05.

**Table 3:** Proximate composition of sausages formulated with grasshopper flour.

Parameters (%)	Formulations ^6^				
	Control	F1	F2	F3	F4
Moisture	65.71 ± 0.08 ^A^	66.84 ± 0.23 ^B^	67.71 ± 0.50 ^C^	66.96 ± 0.07 ^B^	67.06 ± 0.13 ^B,C^
Fat	13.94 ± 0.26 ^C,D^	14.27 ± 0.13 ^D^	13.36 ± 0.25 ^A,B^	13.68 ± 0.02 ^B,C^	13.01 ± 0.07 ^A^
Protein	10.35 ± 0.11 ^A^	15.37 ± 0.27 ^E^	13.34 ± 0.10 ^D^	12.79 ± 0.10 ^C^	12.25 ± 0.17 ^B^
Ashes	2.53 ± 0.10 ^A^	3.41 ± 0.02 ^D^	3.05 ± 0.02 ^B^	3.19 ± 0.04 ^C^	3.21 ± 0.01 ^C^
Salt	1.55 ± 0.09 ^A^	2.64 ± 0.02 ^C^	2.28 ± 0.02 ^B^	2.34 ± 0.02 ^B^	2.38 ± 0.02 ^B^
aw	0.98 ± 0.01 ^A^	0.98 ± 0.01 ^A^	0.98 ± 0.01 ^A^	0.97 ± 0.01 ^A^	0.97 ± 0.01 ^A^
BEFFE ^1^	9.77 ± 0.16 ^A^	13.08 ± 0.23 ^D^	11.83 ± 0.23 ^C^	11.25 ± 0.06 ^B^	10.98 ± 0.23 ^B^
MUFA ^2^	6.70 ± 0.07 ^C^	6.45 ± 0.06 ^B^	6.07 ± 0.10 ^A^	6.16 ± 0.02 ^A^	6.07 ± 0.05 ^A^
PUFA ^3^	1.82 ± 0.02 ^D^	1.68 ± 0.01 ^C^	1.56 ± 0.01 ^B^	1.56 ± 0.01 ^B^	1.49 ± 0.01 ^A^
NPN ^4^	0.72 ± 0.04 ^A^	1.44 ± 0.03 ^C^	1.42 ± 0.04 ^C^	1.09 ± 0.07 ^B^	0.68 ± 0.02 ^A^
SFA ^5^	4.91 ± 0.07 ^C^	4.72 ± 0.06 ^B^	4.46 ± 0.09 ^A^	4.46 ± 0.02 ^A^	4.36 ± 0.03 ^A^

^1^ BEEFE: Bioavailable protein; ^2^ MUFA: Monounsaturated fatty acids; ^3^ PUFA: polyunsaturated fatty acids; ^4^ NPN: Non-protein nitrogen; ^5^ SFA: Saturated fatty acids. ^6^ All values are mean ± standard deviation of three replicates (*n* = 12). Different letters in the same row mean significant differences between formulations at *p* < 0.05.

## Data Availability

The datasets generated for this study are available on request to the corresponding author.
